# *ZmGRAS46* negatively regulates flowering time in maize

**DOI:** 10.1080/21645698.2024.2442158

**Published:** 2024-12-30

**Authors:** Xiaotong Wei, Honglin Zhang, Zhenzhong Jiang, Peng Jiao, Siyan Liu, Shuyan Guan, Yiyong Ma

**Affiliations:** a College of Agronomy, Jilin Agricultural University, Changchun, China; b Joint International Research Laboratory of Modern Agricultural Technology, Ministry of Education, Jilin Agricultural University, Changchun, China; c College of Life Science, Jilin Agricultural University, Changchun, China

**Keywords:** Flowering, GA, maize, yield, *ZmGRAS46*

## Abstract

Flowering time is an important factor limiting the planting area of maize (*Zea may* L.). Gibberellin (GA) can regulate plant flowering time by mediating the GA signaling pathway. This study screened significantly down-regulated gene *ZmGRAS46* by early flowering mutant transcriptomic sequencing (PRJNA788070) in the previous laboratory. The expression pattern analysis of the *ZmGRAS46* gene shows that it has the highest expression level in maize stems. The stem treatment with 200 μmol/L GA_3_ resulted in the lowest expression of *ZmGRAS46* at 3 h. Positive maize plants were obtained through the modified Agrobacterium-mediated genetic transformation of maize. The results showed that overexpression of *ZmGRAS46* delayed the flowering of maize, and gene editing of *ZmGRAS46* made maize blossom earlier. In addition, overexpression of *ZmGRAS46* could increase maize 100-grain weight. This study provides new insights into the molecular mechanism of the *GRAS* gene in regulating plant flowering.

## Introduction

As an essential grain crop, maize ensures the national economy and people’s livelihood. Among them, the flowering period of maize has a vital significance in the growth and development of maize.^1^ In production and development, the flowering time effects maize’s yield, quality, and nutrients.^2^ Maize is a short-day plant with a strong photoperiodic response, so it is economically essential to develop photoperiodic-insensitive maize varieties in temperate regions.^3^ Previous studies showed that the change in flowering time would positively affect the growth and development of maize plants. Therefore, to solve the problem of early or late flowering, it is necessary to study the flowering time of plants. After a long period of evolution, it has been found that the main pathways regulating plant flowering are the photoperiodic pathway,^3^ vernalization pathway,^4^ age pathway,^5^ autonomous flowering time pathway,^6^ temperature pathway,^7^ and GA pathway.^8^ They regulate the expression of florigen genes through different signal transduction pathways in order to achieve the role of regulating flowering.^9^ Through these signal transduction pathways, plants can respond to different environmental stresses and make plants bloom at an appropriate time, which is conducive to plant survival and reproduction.^10,11^

Gibberellin (GA) is a complex hormone in plants, bacteria, and fungi.^12^ GA can regulate the flowering time of plants by mediated GA signaling pathway and affect the flowering time of plants.^13^ Currently, about 130 GA species have been proven to exist, but only a few species with biological activity include Gibberellin A1 (GA_1_) and Gibberellin A_3_ (GA_3_).^14^ The main regulatory factors involved in the GA pathway are *GIBBERELLIN INSENSITIVE DWARF1 (GID1)* and *DELLA*.^15,16^ Among them, *GID1* is transformed from the more prominent hormone-sensitive lipase family (HSLs). A receptor protein that binds GA.,^1,17^DELLA a GA signal receiver with the conserved domain. This growth inhibitory factor negatively regulates *FT* expression and a central regulator of the GA pathway.^18^ When GA concentration is low, GA and DELLA combine to inhibit the initiation of transcription of florigenes and inhibit flowering.^19^ At high GA concentrations, GA binds to *GID1* to form a complex, which binds to DELLA stably. DELLA is degraded under the action of SLEEPY1(SLY1) protein, activating the initiation of flower formation and promoting plant flowering.^20^ Yang et al.^21^ demonstrated in *Arabidopsis* that DELLA family members can interact with *bHLH48* and *bHLH60* to change the expression of *FT* and positively mediate the GA pathway to regulate plant flowering. Moreover, GA can change the inhibitory effect OF DELLA on flowering regulatory genes such as *LEAFY(LFY)* and *SUPPRESSOR OF CONSTANS1(SOC1)*, thus promoting the flowering of *Arabidopsis*. Liu et al.^22^ showed that *GAMYB*, as the primary activator of the GA signaling pathway in rice, regulates the expression of GA-induced genes in plant anthers and affects the development of anthers. Wang et al.^23^ showed that SAW1 encodes CCCH-type zinc finger protein, which acts on the promoter domain of *GA20ox3*, mediates the GA signaling pathway, induces specific expression in plant anther, and positively regulates GA homeostasis.

GRAS (gibberellic acid insensitive, repressor of GAI, and scarecrow) family is a unique transcriptional factor that plays a vital role in plant growth and development.^24^ It is named after the first three transcription factors discovered: Gibberellic acid insensitive (GAI), Repressor of *GA1–3* mutant (*RGA*), and *Scarecrow (SCR)*.^25^ Through evolutionary analysis of GRAS family members of model plants *Arabidopsis* and rice, the family was divided into 8 subfamilies: Scarecrow-like 3 (SCL3), Lilium longiflorum SCR-like (LISCL), Short-root (SHR), Phytochrome A signal transduction 1 (PAT1), DELLA, SCR, Lateral suppressor (LS), and Hairy meristem (HAM).^25–27^ There is a conserved DELLA family in the GRAS family, and it has been proved that DELLA, as the central repressor of the GA signaling pathway, can interact with many transcriptional regulatory factors distributed on the leaves and stem tips of plants to regulate the flowering time of plants.^28,29^ The GA signaling pathway regulates flowering mainly by influencing the expression of *FT*. GA can stimulate the transcription activity of *FT* to make plants bloom under long-day conditions. In contrast the transcription activity of *FT* is inhibited in GA mutants, suggesting that *DELLA* regulates plant flowering by regulating the expression of *FT*.^30^ Jutarou et al.^31^ found that *DELLA* is a co-activator of *GAI ASSOCIATED FACTOR 1 (GAF1)*. Four inhibitory factors, *EARLY FLOWERING3 (ELF3)*, *SHORT VEGETATIVE PHASE (SVP)*, *TEMPRANILLO1 (TEM1)*, and *TEM2*, are the target genes of *GAF1*.^31^ *DELLA*, promote the expression of *FT* by inhibiting four flowering inhibitors and mediates GA signaling pathway to regulate the flowering time of plants.^30^ In another study of plants, it was found that *SLR1* is a repressor of GA signal, which is more expressed during pollen development. It inhibits *GID1* degradation through N-terminal phosphorylation, thus inhibiting GA signal transduction.^32^ RGL2 is one of the DELLA proteins.^32^ Studies have shown that *RGL2* regulates flower development, mainly regulating stamen growth and anther division, and dramatically influences plant growth.^33^ *GRAS* genes rarely have intron insertion in evolution, and the DELLA family gene structure is relatively simple and conserved. The function of the DELLAs protein has been widely studied in recent years, but *ZmGRAS46* in the DELLA family has yet to be studied.

In this study, the significantly down-regulated gene *ZmGRAS46* was screened by the transcriptome sequencing of the early-flower mutant in the previous laboratory (PRJNA788070). Overexpressed and CRISPR/Cas9 vectors were constructed, and maize was transformed by an improved Agrobacteria-mediated method. The expression pattern and regulatory relationship were analyzed to further clarify the biological function of the *ZmGRAS46* gene.

## Result

### *Bioinformatics prediction and expression pattern analysis of* ZmGRAS46 *gene*

The *ZmGRAS46* (Zm00001d044065) gene was screened from the laboratory early flowering mutant sequencing library (PRJNA788070). Bioinformatics analysis showed that the number of amino acids encoded by the *ZmGRAS46* gene sequence was 475, the relative molecular weight was 50,563.04, the theoretical isoelectric point was 5.55, the chemical formula was C2233H3462N652O661S17, and the total number of atoms was 7025. The aliphatic amino acid index was 83.39 (see Figure S1A). The protein is a hydrophilic protein (see Figure S1B). The secondary structure of this gene is composed of 53.26% α helix, 9.89% extended chain, 4.21% β fold, and 32.63% random curl (see Figure S1C). The phylogenetic tree analysis showed that ZmGRAS46 was closely related to homologous protein sequences in sorghum (see Figure S1D). The tissue expression pattern of genes was analyzed, and the results showed that the expression of *ZmGRAS46* was highest in stems. The relative expression of *ZmGRAS46* was lowest in male and female ears at the tasseling stage of the maize growth period (0.16 and 0.15) (Figure S2).

**Figure 1.**
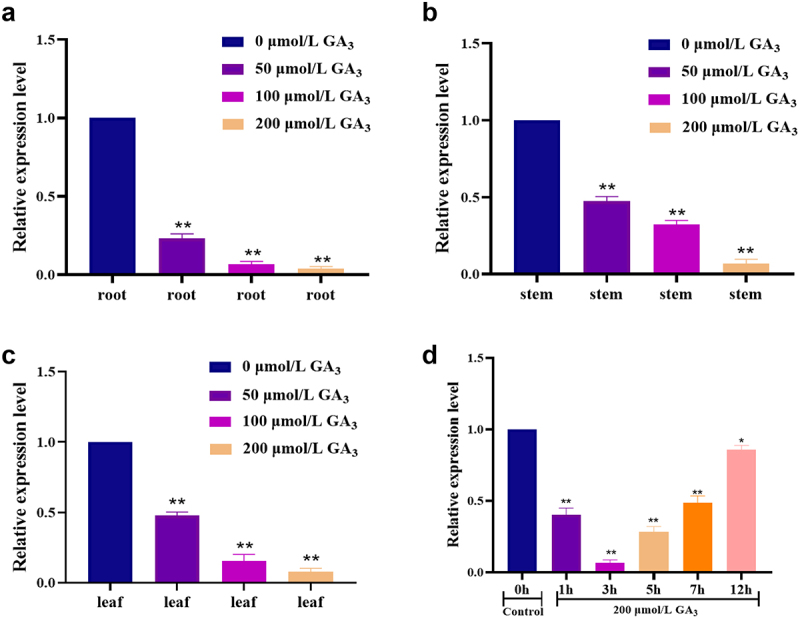
Analysis of relative expression of *ZmGRAS46* after GA_3_ treatment. (a–c) analysis of expression patterns of *ZmGRAS46* in root and leaves under different GA3 concentrations. (d) Analysis of *ZmGRAS46* gene expression pattern in maize stems treated with 200 μmol/L GA_3_ for 1, 3, 5, 7, 12 h. Using student’s t-test, asterisks indicate statistically significant differences (*p < .05; **p < .01). Data are shown as mean ± SD from three independent experiments.

Since the *ZmGRAS46* gene belongs to the DELLA subfamily, a critical inhibitory factor in the GA pathway,^34,35^ the expression level of the *ZmGRAS46* gene in maize under different GA_3_ concentrations was investigated (see Figure 1). qRT-PCR of maize’s roots stems, and leaves in the three-leaf stage after GA_3_ treatment was carried out (see Figures 1a–c). The results showed that the expression of *ZmGRAS46* in maize root and leaves was the lowest when GA_3_ concentration was 200 μmol/L, so the stems were treated with 200 μmol/L GA_3_. The results showed that the expression of *ZmGRAS46* was the lowest at 3 h, indicating that the maize was treated with 200 μmol/L GA_3_ for 3 h (see Figure 1d). Exogenous application of gibberellin was used to detect how *ZmGRAS46* gene responds to gibberellin signal. It was found that with the increase of gibberellin content, *ZmGRAS46* gene expression decreased, forming a negative feedback regulatory mechanism. Therefore, 200 μmol/L GA_3_ concentration at the lowest expression level of *ZmGRAS46* gene was used to continue the experiment, and the specific response time was determined to be 3 h.

### Improvement of maize genetic transformation system and acquisition of transgenic plants

**Figure 2.**
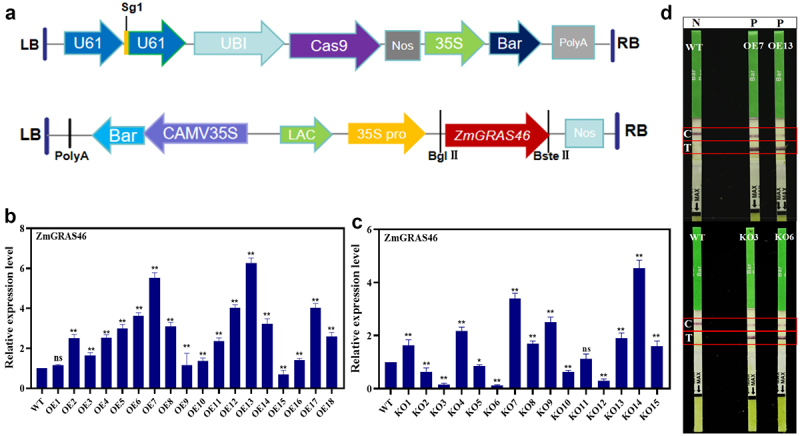
Molecular detection of transgenic positive plants. (a) Structure of pCXB053-ZmGRAS46 and pCAMBIA3301-ZmGRAS46 vector. LB: left border of T-DNA; RB: right border of T-DNA; U6: U6 promoter; NOS: NOS terminator; 35S: CaMV 35S promoter; BAR: bar selection marker gene; PolyA: PolyA terminator. (b) Expression of *ZmGRAS46* in maize gene-edited plants, WT stands for wild type strain, OE1–18 is the expression level of *ZmGRAS46* gene in overexpressing strains. (c) Expression of *ZmGRAS46* in maize overexpression plants, WT stands for wild type strain, KO1–15 is the expression level of *ZmGRAS46* gene in the knockout strain. (d) Bar quick test paper experiment. Control line (C), test line (T), negative (N), positive (P). Using Student’s t-test, asterisks indicate statistically significant differences (*p < .05; **p < .01; ns = not significant). Data are shown as mean ± SD from three independent experiments.

The higher the callus induction rate of maize genetic transformation, the better the genetic transformation effect. Through the comparison of the callus induction rate of the immature embryos, the best-inbred line H208 was selected for the follow-up test (see Table S1, Figure S3). The changes in dry weight, fresh weight, cell morphology, phenotype, and endogenous auxin synthesis factors of maize callus under red light, blue light, white light, dark, and red blue light irradiation were investigated (see Figures S4-S7). The results showed that red and blue light promoted the accumulation of biomass in maize callus (see Figure S5), cell growth, and synthesis of *ZmYUCCA2*, *ZmYUCCA6*, and *ZmVT2* (see Figure S6), thereby increasing auxin content, promoting the differentiation of maize callus, and improving the conversion efficiency. Transgenic plants with overexpression of the *ZmGRAS46* gene (OE1–18) and knockout of the *ZmGRAS46* gene (KO1–15) were obtained by using this optimized system (see Figure 2). The target information of the knockout strain is shown in Figure S7. The plasmid profile is shown in Figure 2a. QRT-PCR detection of transgenic plants was carried out, and OE7, OE13, KO3, and KO6 were selected for subsequent tests (see Figures 2b, c). Bar test strips were performed for OE7, OE13, KO3, and KO6 transgenic strains. The results showed that the WT control line (C) showed color, the test line (T) did not show color, and the positive plant C and T lines were visible to the naked eye, which proved that the transgenic strain was successfully obtained (see Figure 2d).

### Phenotypic differences in the flowering stage of transgenic maize

**Figure 3.**
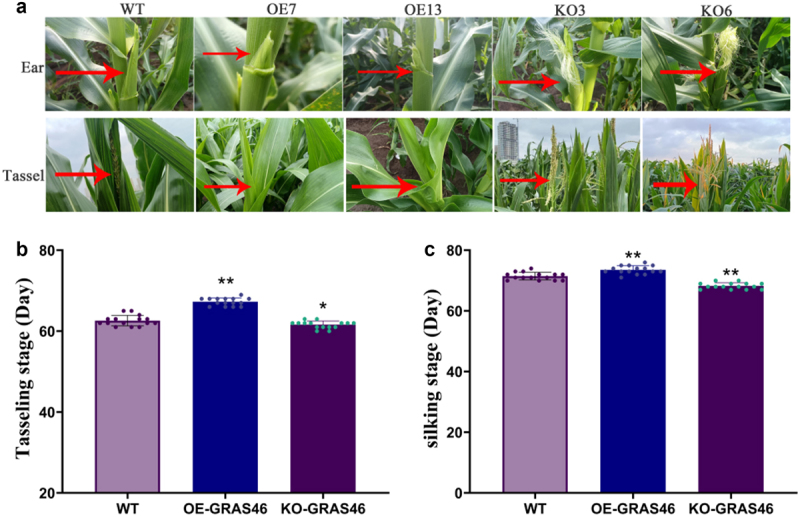
Analysis of flowering phenotype and flowering time of transgenic maize plants. (a) Phenotype of genetically modified maize during tasseling and silk emergence stages; (b) time statistics of tasseling period in genetically modified maize; (c) time statistics of silk production stage in transgenic maize; using student’s t-test, asterisks indicate statistically significant differences (*p < .05; **p < .01). Data are shown as mean ± SD from three independent experiments.

In order to detect the effect of *ZmGRAS46* overexpression and gene editing on maize flowering, stable T_2_ generation OE7, OE13, KO3, and KO6 transgenic seeds were sown in transgenic experimental fields, and the flowering phenotypes were recorded and analyzed (see Figure 3a). The results showed that in the WT, the tasseling and silking time were 62.6 and 71.47, respectively; The time of tasseling and silking of overexpressed transgenic plants were 67.27 and 73.53, respectively; The tasseling and silking time of gene-edited plants were 61.60 and 68.27, respectively. In other words, in both flowering stages, overexpressed transgenic plants were later than WT and gene-edited plants were earlier than WT (see Figures 3b, c). It was preliminarily determined that *ZmGRAS46* negatively regulated the flowering process of maize.

### Determination of physiological and biochemical indexes of transgenic maize plants treated with GA_3_

**Figure 4.**
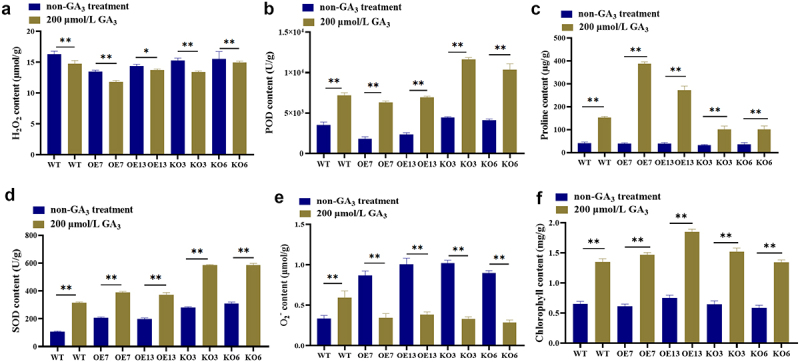
Analysis of physiological and biochemical indicators of transgenic maize plants before and after GA_3_ treatment. (a) H_2_O_2_ content; (b) POD enzyme activity; (c) proline content; (d) SOD enzyme activity; (e) O_2_^−^ content; (f) chlorophyll content. Using student’s t-test, asterisks indicate statistically significant differences (* *p* < .05; ** *p* < .01). Data are shown as mean ± SD from three independent experiments.

Without GA_3_ processing conditions, there was no significant difference in proline and chlorophyll content between WT and transgenic strains; The SOD enzyme activity, and O_2_^−^ content of the transgenic strain were higher than those of the WT; The POD enzyme activity of the overexpression strain was significantly lower than that of the WT, while the knockout strain was significantly higher than that of the WT; The H_2_O_2_ content of overexpressing plants was significantly higher than that of the WT, while the H_2_O_2_ content of knockout strains showed no significant difference compared to the WT. The leaves of four-leaf maize were sprayed with 200 μmol/L GA_3_, and the physiological and biochemical indexes of the leaves were measured at 3 h. The results showed that after spraying GA_3_, POD (see Figure 4b), Pro (see Figure 4c), SOD (see Figure 4d), and total chlorophyll content (see Figure 4f) in WT, OE7, OE13, KO3 and KO6 plants could be increased. Reduces H_2_O_2_ content in WT, OE7, OE13, KO3, and KO6 plants (see Figure 4a). The O_2_^−^ content in OE7, OE13, KO3, and KO6 plants was significantly reduced after GA_3_ was applied (see Figure 4e). The above results indicated that spraying 200 μmol/L GA_3_ benefited to plant growth.

### Detection of flowering-related gene expression in transgenic maize

**Figure 5.**
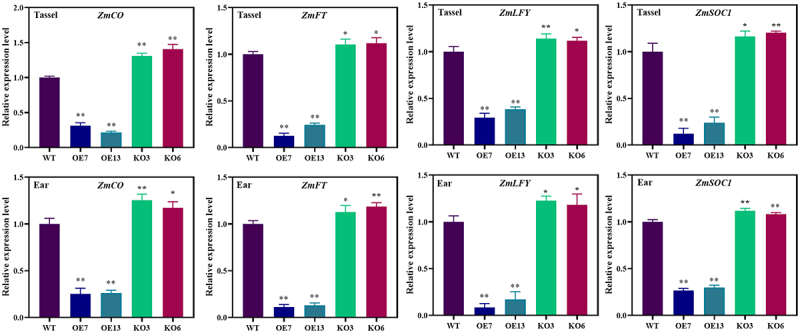
Expression of flowering-related genes in transgenic maize of generation. (a–d) the expression levels of *CO*, *FT*, *LFY*, and *SOC1* genes in tassel. (E-H) the expression levels of *CO*, *FT*, *LFY*, and *SOC1* genes in ear. Using Student’s t-test, asterisks indicate statistically significant differences (*p < .05; **p < .01). Data are shown as mean ± SD from three independent experiments.

In order to study the effect of *ZmGRAS46* on flowering, the expression levels of flowering-related genes in male and female maize ears were measured by qRT-PCR. The results showed lower *CO*, *FT*, *SOC1*, and *LFY* expressions in OE7 and OE13 plants than those in WT, KO3, and KO6 plants (see Figure 5). These results indicated that overexpression of *ZmGRAS46* inhibited the expression of *CO*, *FT*, *SOC1*, and *LFY* genes. The expression of *CO*, *FT*, *SOC1*, and *LFY* genes in KO3 and KO6 strains was significantly higher than that in WT. The *ZmGRAS46* gene is a key gene for flowering, and both overexpression and knockout of this gene affect the expression of flowering related genes. However, overexpression plants have a greater impact on the expression of flowering genes. These results indicated that overexpression of *ZmGRAS46* delayed flowering by inhibiting the expression of flowering-related genes, which was consistent with the results of the maize phenotype.

### Analysis of agronomic traits of transgenic maize

**Figure 6.**
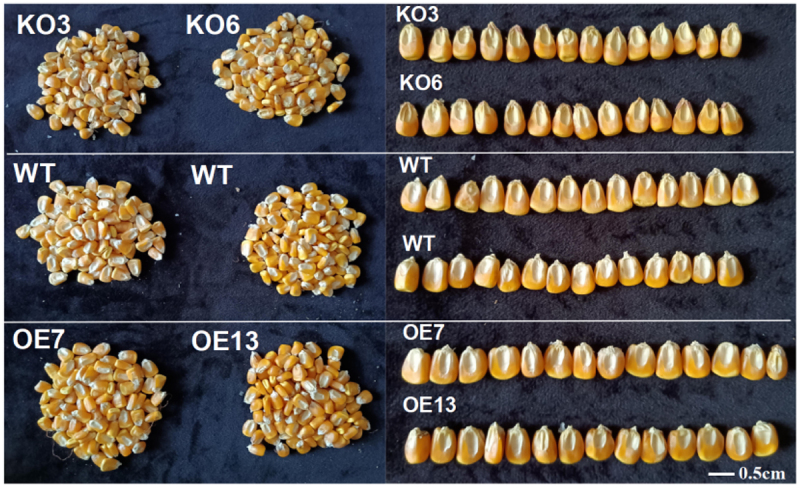
Analysis of yield related traits in T_2_ generation transgenic maize plants.

Statistical analysis was conducted on the yield-related traits of stable T_2_ transgenic maize. The results showed that the 100-grain weight of overexpressed maize plants was more significant than that of WT plants than that of gene-edited maize plants. The ear size of gene-edited maize plants was more significant than that of overexpressed maize plants in WT, but there was no significant difference in axis size, grain length, and row number of ears. The results indicated that overexpression of *ZmGRAS46* could increase the 100-grain weight of maize (see Figure 6).

## Materials And Methods

### Plant material

Maize early flowering mutant M12164 was obtained by EMS mutagenesis from the Plant Biotechnology Center, Jilin Agricultural University. The seeds of maize inbred lines H202, H205, H208, H2015, and H2017 were sown in the experimental field of the Plant Biotechnology Center of Jilin Agricultural University.

### *Bioinformatics analysis of* ZmGRAS46 *Gene*

In the NCBI lookup *ZmGRAS46* gene sequence, the gene CDS area is translated into the amino acid sequences, through online software ProtParam (https://web.expasy.org/protparam/.), SOP-MA (https://npsa-prabi.ibcp.fr/cgi-bin/npsa_aut-omat.pl?page=npsa_sopma.html.), ProtScale (http://w-ww.cbs.dtu.dk/services/NetPho-s/.), SWISS – MODEL (https://swissmodel.expasy.org/.) for *ZmGR-AS46* gene sequences of physical and chemical properties, personal relationship forecast analysis on water, protein structure and evolutionary tree.

### *Tissue expression analysis of* ZmGRAS46 *Gene in maize*

The template used for Quantitative Real-time PCR (qRT-PCR) was the cDNA of maize root, stem, leaf, female, and male ear, and the internal reference genes were *ZmActin1* and *ZmGRAS46* genes as the target genes for qRT-PCR analysis. All primer sequences in this experiment are shown in Table S3. The experiment was repeated three times for each sample, and the relative expression of the gene was calculated by the formula 2^−△△Ct^ after the reaction.

### *Expression analysis of the* ZmGRAS46 *Gene in maize treated with GA_3_*

The seeds of laboratory maize early flowering mutant M12164 were germinated under water-soaked germination paper. After a week of dark culture, the seeds germinated and took root. When it was placed at 25°C under 16 h light/8 h darkness to grow to tri leaf stage, 50 μmol/L, 100 μmol/L and 200 μmol/L GA_3_ (1:1000) were added to the culture bottle for hormone treatment, respectively. Maize roots, stems and leaves treated for 0 h, 1 h, 3 h, 5 h, 7 h, and 12 h were sampled, and each sample was repeated three times. All primer sequences in this experiment are shown in Table S3. After the reaction, the relative expression of the gene was calculated using formula 2^−△△Ct^.

### Plasmid construction and genetic transformation

Design gene-specific primers using primer design software. Clone *ZmGRAS46* using polymerase chain reaction (PCR) technology. The protein coding region of ZmGRAS46 was cloned by PCR using specific primers, digested with BglII and BstEII enzymes, and then ligated to a vector containing the CaMV 35S promoter (pCAMBIA3301). Design appropriate sgRNAs using software (https://zlab.bio/guide-design-resources.) based on the *ZmGRAS46* sequence to guide Cas9 protein onto the *ZmGRAS46* sequence. Clone the sgRNA and Cas9 coding sequences into the pCXB053 vector. The pCAMBIA3301-GRAS46-Bar and pCXB053-GRAS46-Bar recombinant vectors were transformed into Agrobacterium EHA105 strain using an improved genetic transformation method, and then transformed into WT plants.

### Obtaining maize callus

First, select maize that has been pollinated for 7–15 days, thoroughly soak it in 75% alcohol for disinfection on a clean bench, and sterilize it for 15 minutes. After soaking, remove the maize, put it in 5% NaClO, sterilize it for 10 minutes, rinse it with sterile water, and rinse it repeatedly 4–5 times. Then, one-third of the upper part of the maize kernels was cut off with a sterilized blade, and the young maize embryos were taken out. Finally, the young maize embryos were cultured in MS medium in the dark, and the embryos were cut off irregularly during the culture, and a stable callus would be formed in about 35 days.

### Selection of optimal maize inbred lines

The best-inbred lines were selected as the research object to study the maize-inbred lines H202, H205, H208, H2015, and H2017. In this study, the immature embryos of these five inbred lines were cultured in MS medium, and their growth was observed and recorded. The inbred lines with the best callus induction were selected for subsequent experiments.

### Maize callus was cultured in different auxin media

In order to study the effect of auxin on callus induction under different light treatments, in this experiment, the MS medium with 1 mg/ml of 2,4-D, 1 mg/ml of NAA, and 1 mg/ml of IAA and the MS medium without exogenous auxin were selected. The immature embryos of maize inbred line H208 were cultured in the above media. The callus induction was observed under red light, blue light, red and blue light, white light, and darkness.

### Maize embryogenic callus was cultured under different light treatments

In order to study the effect of different light qualities on callus differentiation, maize embryonic callus was cultured in an MS medium. In this experiment, the callus was cultured in the dark for 14 hours, including white light, red light, blue light, red and blue light co-irradiation, and culture in complete darkness. In this experiment, the fresh weight and dry weight of the callus cultured for seven days were weighed, and the phenotypic changes of the callus were analyzed. The cell sections of the callus cultured for fifteen days were observed, and the phenotypic changes of callus were recorded. The callus cultured for fifteen days was observed by cell section, and ImageJ measured the relative cell diameter of the cell section, and the growth of cells was recorded by comparing the relative diameters.

### Determination of endogenous auxin-related gene expression in maize callus under different light treatments

Auxin plays a vital role in callus induction and differentiation. Our laboratory’s transcriptome sequencing database selected the genes ZmYUCCA2, ZmYUCCA6, ZmTAR2, ZmVT2, and ZmAUS2, which mainly affect the change of auxin content in maize. Because different light treatments have different effects on the expression of auxin in the callus, the expression of the above genes was studied by qRT-PCR in this experiment. All primer sequences in this experiment are shown in Table S3.

### Detection of transgenic maize plants

Test with Bar quick test strip (produced by Oil Crops Research Institute, Chinese Academy of Agricultural Sciences, batch No. 20230815). If the concentration of transgene in the sample is more significant than 0.1%, the control line (C) and test line (T) show color, and the result is positive (P). Conversely, the C shows color, the T does not, and the result is negative (N). With wild-type (WT) maize as the control group and positive maize as the experimental group, qRT-PCR was carried out on T_2_ substituted *ZmGRAS46* gene maize plants, and the optimal positive plants were selected as the primary research object of the subsequent test.

### Analysis of flowering phenotype of transgenic maize plants

Transgenic and WT maize seeds were sown in the transgenic fields of Jilin Agricultural University, and the flowering phenotypes of maize were statistically analyzed and recorded when the maize entered the tasseling stage. OE-ZmGRAS46#7, OE-ZmGRAS46#13, KO-ZmGRAS46#3 and KO-ZmGRAS46#6 were sown with 50 plants respectively, and 100-grain weight, ear length, axis diameter, grain length and row number of ears were investigated at the mature stage of maize.

### Determination of physiological and biochemical indexes of transgenic maize plants treated with Ga_3_

The maize was treated with 200 μmol/L GA_3_ at the four-leaf stage, and the leaves were sampled after three hours of spraying. The contents of superoxide dismutase (SOD), peroxidase (POD), proline, chlorophyll, hydrogen peroxide (H_2_O_2_), and superoxide anion (O_2_) were measured. Kits were purchased from Beijing Boxbio Science ＆ Technology Co. Ltd.

### Detection of flowering-related gene expression in transgenic maize

Male and female ear samples of T_2_ generation positive maize and WT maize were sampled for RNA extraction and then reverse-transcribed into cDNA for qRT-PCR. All primer sequences in this experiment are shown in Table S3.

### Data analysis

In this study, each experiment was repeated three times, and the graph was drawn by GraphPad Prism 8.0. The values are means (± SE) of three biological replicates. Data are means ± SE, ns indicates that the difference is not significant, * and ** indicate a significant difference at *p < .05* and *p < .001* probability, respectively.

## Discussion

GRAS family has been studied in grapes,^36^ cassava,^37^ bananas,^38^ oats,^39^ quinoa,^40^ cucumber,^41^ and other plants, but more studies in maize and its mechanism still need to be determined. Therefore, this experiment selected *ZmGRAS46*, a family member, to explore its role in maize. The physicochemical properties, hydrophilicity, and protein structure of the gene were analyzed by bioinformatics (see Figure S1). The function of *ZmGRAS46* is studied according to the predicted direction. In the Salvia miltiorrhiza study, most *SmGRAS46* genes showed significant responses to GA, suggesting they may play an essential role in the GA signaling pathway. In the study of tomatoes, it was found that *SlGRAS24* regulated the agronomic traits of tomatoes by regulating the signal transduction of GA and auxin.^42^ GA stress can affect the expression of four *PgGRAS* genes of the DELLA family, thus affecting the growth and development of ginseng hair roots.^43^ The results that *ZmGRAS46* can affect the growth and development of maize in response to GA in this experiment are consistent with the results that the *GRAS* gene can respond to GA. The maize was treated with GA_3_, and the optimal concentration and time were selected to preliminarily verify that *ZmGRAS46* had a specific effect on the flowering time of maize and confirmed that the gene was sensitive to GA_3_, which provided a theoretical basis for subsequent experiments (see Figure 1).

Mostafa et al.^44^ have shown that genotypes and auxin influence callus growth. Genotype is significant for the induction of maize callus, and high-quality maize genotypic explants are more conducive to introducing type II callus. They can be passed for a long time to form regenerated plants through the embryoid pathway.^45,46^ The extraction rate of the callus of the best-inbred line H208 was 96.2%, a very high-quality experimental material (see Table S1 and Figure S3). Using H208 as experimental material, the effects of different auxins on callus differentiation were studied (see Table S2 and Figure S4). The results showed that adding exogenous auxin was only beneficial to the induction of callus but inhibited the differentiation of callus. We hypothesized that callus differentiation might be regulated by endogenous auxin synthesis, which was also verified in later studies. Different light treatments, showed that it was more suitable for inducing callus in the dark (see Figure S5). Compared with other light treatments, callus can form dense callus in the dark, be stably subcultured, and be regenerated through buds, which provides stable experimental materials for the subsequent genetic transformation of maize. Light plays a specific role in the metabolic activity of plants, and the study of Johkan et al.^47^ showed that the growth state of callus may be related to light. Nhut et al.^48^ showed that yellow light can accelerate callus growth. By observing the phenotypic changes of plant differentiation under different light treatments, it was found that red light and blue light could accelerate the differentiation of maize callus; red light and blue light could promote the growth of leaves and the formation of adventitious roots. Compared with white light, this study suggested that red and blue light could accelerates the formation of adventitious roots and buds, making the maize callus differentiate faster. Different light treatments can significantly affect cell viability. To further explore how red and blue light accelerate plant differentiation, we took the most significant section of the callus, and its size changed significantly (see Figure S7). Studies have found that red light promotes cell growth and blue light promotes cell division, especially in red and blue light. The number of cells is significantly increased and relatively large, indicating that red light and blue light can accelerate cell growth. The application of exogenous auxin could not promote callus differentiation, so we studied the synthesis of endogenous auxin in callus. Auxin can promote the growth and development of plants, and different light treatments will also affect the synthesis rate of endogenous auxin. QRT-PCR analyzed the expression of endogenous auxin in maize. The results showed that compared with white light, red light, blue light, and red blue light accelerated the expression of *ZmYUCCA2*, *ZmYUCCA6*, *ZmVT2*, and *ZmAUS2* (see Figure S6). In conclusion, the best light treatment was found to promote the differentiation of callus, improve the differentiation rate of callus, and provide a reasonable basis for accelerating the genetic transformation of maize.

POD and SOD are the main enzymes in the plant antioxidant system, and their activity levels can reflect the degree of the plant affected by external stress. POD is an enzyme that clears H_2_O_2_. The content of free radicals in plants is maintained steadily through synergistic action. Under untreated conditions, the POD enzyme activity of transgenic lines was increased, indicating that both OE-ZmGRAS46 (delayed flowering) and KO-ZmGRAS46 (promoted flowering) genes caused changes in the plant’s oxidative system (see Figure 4). This indirectly suggests that the knockout and overexpression of this gene may have affected the expression of antioxidant enzyme-related genes, thereby affecting enzyme activity. Under GA_3_ treatment conditions, the POD enzyme activity of the KO-ZmGRAS46 strain was significantly higher than that of the WT, indicating the production of many oxidative factors during the process of promoting flowering (see Figure 4b). The POD enzyme activity of OE-ZmGRAS46 under GA_3_ treatment conditions was not significantly different from that of the WT but still higher than that of the overexpression strain without GA_3_ treatment. The induction of GA_3_ did not induce an oxidative stress response in overexpressing plants, and the ZmGRAS46 gene may be involved in expressing antioxidant enzyme-related genes. SOD is an enzyme that clears O_2_^−^. Under conditions without GA_3_ treatment, it was found that SOD enzyme activity was positively correlated with O_2_^−^ content (see Figure 4d). The overexpression and knockout of the ZmGRAS46 gene not only cause the production of oxidative factors but also play a protective role in the antioxidant enzyme system in the body, to some extent offsetting the production of O_2_^−^ content, thereby achieving a steady state of free radicals in the plant (see Figure 4e).

There is much research on regulating GA on seed vigor, seedling development, and drought resistance in maize, but how to affect the flowering period of maize by exogenous application of GA is still a problem.^49–51^ In this study, the effect of *ZmGRAS46* in regulating plant flowering time was verified again by establishing the overexpression and gene editing vector of *ZmGRAS46* (see Figure 2). However, at present, only the regulatory effect of *ZmGRAS46* on flowering time has been verified, and the function of *ZmGRAS46* mediating GA signaling pathway to regulate maize flowering time needs to be further studied, and whether exogenous application of GA can change maize flowering time needs to be further discussed. Crops need to achieve high yield. Gautam V et al.^52^ have shown that early flowering mutants obtained by mutagenesis of rice by the gamma-ray and EMS could affect rice yield, indicating that the early maturation of crops has specific research significance. Wheat yields are influenced by environmental, management, and genotypic factors. To cope with climate changes, farmers must have different varieties on hand, such as early flowering, mid- and late-flowering, and late-flowering varieties, and plant according to seasonal conditions.^53^ Hussin et al.^54^ showed that *MADS-box* transcription factors can regulate fruit ripening, *SOC1* in *MADS-box* can mediate plant flowering, and this family also plays a role in influencing yield. In the investigation and analysis of maize agronomic traits in this experiment, it was found that *ZmGRAS46* had an effect on maize yield, but whether the interaction between *ZmGRAS46* and *ZmMADS62* regulated the change of maize yield has not been studied (see Figure 6 and Table 1). It only laid a particular theoretical foundation for future studies on the effect of *ZmGRAS46* on plant yield.

**Table 1.** Agronomic traits analysis of maize transgenic with *ZmGRAS46* gene

Number	Hundred grain weight/g	Ear length/cm	Axis thickness/cm	Grain length/cm	Number of rows per spike
WT-1	25.48±0.62	13.23±0.35	3.62±0.18	0.75±0.27	14
WT-2	25.12±0.13	12.89±0.14	3.54±0.32	0.78±0.32	14
WT-3	25.32±0.33	12.94±0.42	3.48±0.43	0.79±0.29	14
OE7-1	27.16±0.24**	11.32±0.46**	3.56±0.23	0.77±0.33	14
OE7-2	26.89±0.42**	11.21±0.11**	3.42±0.36	0.75±0.42	14
OE7-3	26.92±0.31**	11.12±0.42**	3.37±0.44	0.76±0.38	14
OE13-1	25.82±0.56*	11.05±0.24**	3.60±0.11	0.76±0.27	14
OE13-2	26.12±0.28*	11.23±0.32**	3.57±0.26	0.77±0.33	14
OE13-3	25.42±0.37*	11.19±0.13**	3.62±0.17	0.78±0.41	14
KO3-1	23.12±0.29**	13.52±0.53*	3.64±0.42	0.73±0.32	14
KO3-2	23.34±0.45**	14.03±0.14**	3.59±0.24	0.76±0.29	14
KO3-3	23.52±0.12**	13.74±0.23*	3.62±0.22	0.77±0.34	14
KO6-1	24.37±0.22*	13.82±0.34*	3.65±0.28	0.72±0.35	14
KO6-2	24.22±0.36*	13.93±0.28*	3.63±0.32	0.74±0.43	14
KO6-3	24.18±0.47*	13.68±0.18*	3.61±0.16	0.74±0.39	14

### Limitations of the study

Due to the relatively long breeding time of maize itself, a lot of time was spent on the genetic transformation of maize and the acquisition of positive plants in the early stage. The functional verification of positive maize plants in this study is not comprehensive enough. The upstream and downstream regulatory mechanism of *ZmGRAS46* gene needs to be explored, and the change trend of yield-related genes needs to be further discovered.

## Supplementary Material

supplementary_material clean.docx

## Highlights

- In this laboratory, the maize inbred line material was modified by an Agrobacterium-mediated method, the genetic transformation of maize was realized, and finally, transgenic plants were obtained.
- Field phenotypic analysis of transgenic plants showed that overexpression of *ZmGRAS46* delayed the flowering of maize, while gene editing of *ZmGRAS46* made maize blossom earlier.
- Overexpression of *ZmGRAS46* can increase the 100 grain weight of maize.
