# Supplementary material for: ZmGRAS46 negatively regulates flowering time in maize
Source: GM Crops Food. 2024 Dec 30;16(1):83–96. doi: 10.1080/21645698.2024.2442158 (PMC11702965; doi:10.1080/21645698.2024.2442158)
Supplement: supplementary_material clean.docx [file KGMC_A_2442158_SM1960.docx]

**Supplemental information**


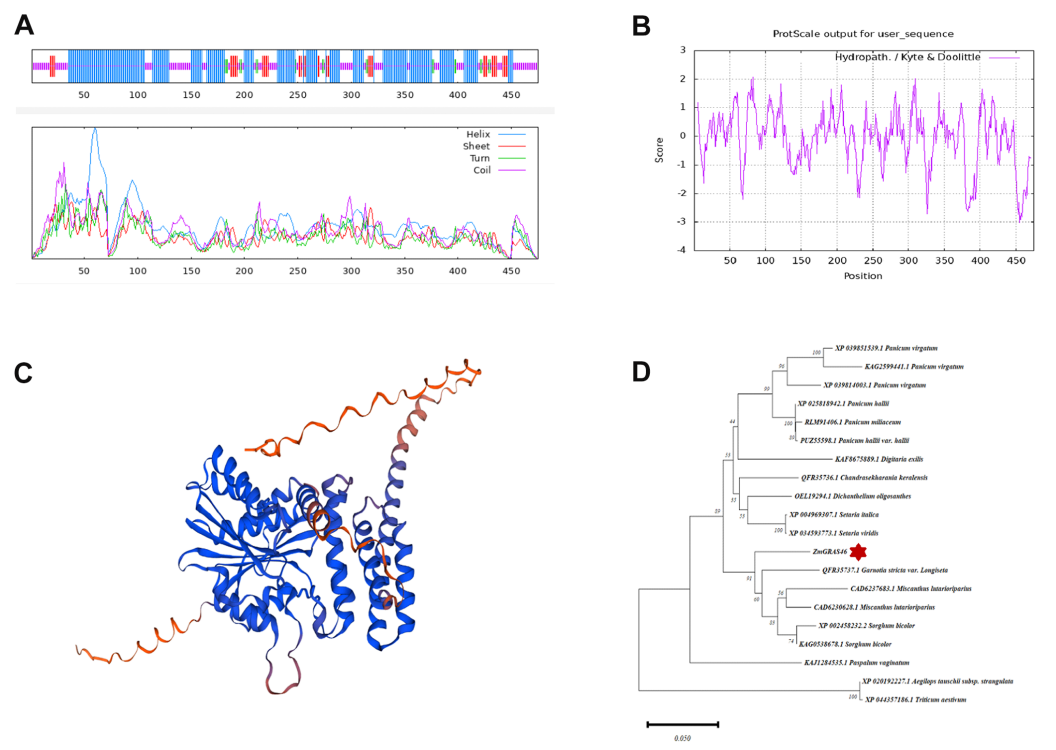


**Fig S1.** Bioinformatics analysis of *ZmGRAS46* gene. (A) Online analysis of the affinity of the amino acid sequence encoding *ZmGRAS46.* (B) Secondary structure analysis of the amino acid sequence encoding *ZmGRAS46.* (C) Tertiary structure analysis of the amino acid sequence encoding *ZmGRAS46.* (D) Evolutionary tree analysis of *ZmGRAS46* gene encoding protein.


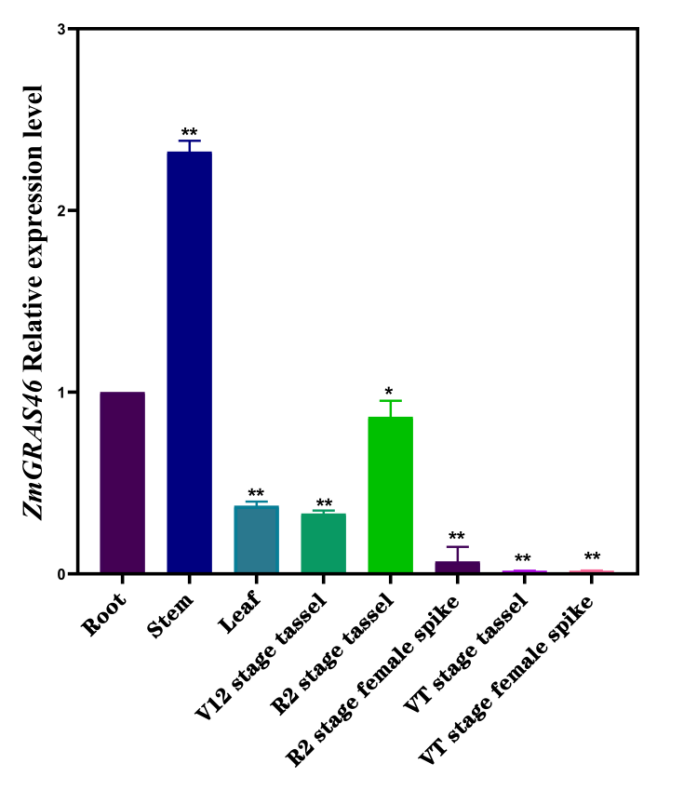


**Figure S2.** Analysis of expression patterns of *ZmGRAS46* in different parts of maize.* P < 0.05 and ** P < 0.01

**Table S1.** Embryogenic callus produced by different maize inbred lines

| Inbred lines | Induction rate of Type II callus (%) | Callus growth |
| --- | --- | --- |
| H208 | 96.2%±0.23 | golden yellow, loose, Callus is the most |
| H205 | 87.5%±0.19 | Milky white or golden yellow, looser and more callus |
| H2015 | 78.7%±0.37 | Pale yellow, dense, less callus |
| H2017 | 78.2%±0.42 | Pale yellow, dense, less callus. |
| H202 | 43.3%±0.13 | White and transparent or yellow, hard, with few callus. |

**Table S2.** Growth of embryonic callus after adding different exogenous auxin

| Type | Callus growth |
| --- | --- |
| 2,4-D | The time of callus formation was the earliest, and the number of type Ⅱ callus was the largest. |
| NAA | The time of callus formation was moderate, and the number of type Ⅱ callus was average. |
| IAA | The time of callus formation was late, and the number of type Ⅱ callus was average. |
| Blank | The time of callus formation is the latest, and the number of type Ⅱcallus is small. |


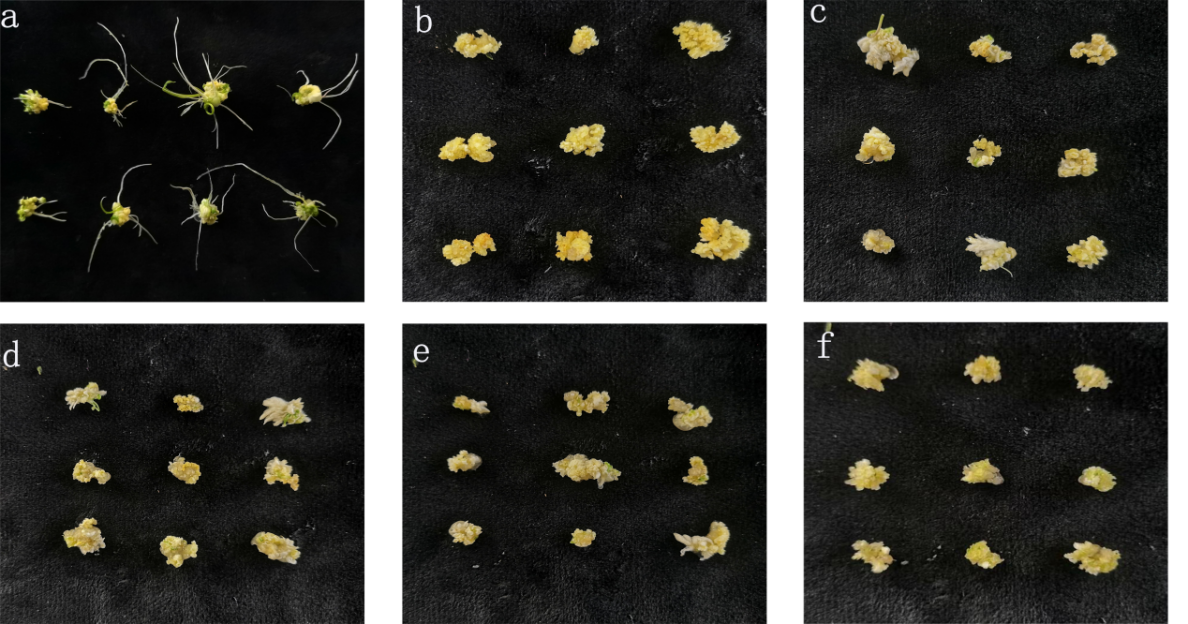


**Figure S3.** The growth of callus under different light treatments in the presence or absence of 2,4-D medium. (A) Culture under light without adding 2,4-D; (B-F) Growth of callus in the dark, white light, blue Light, red light, red and blue light.

**
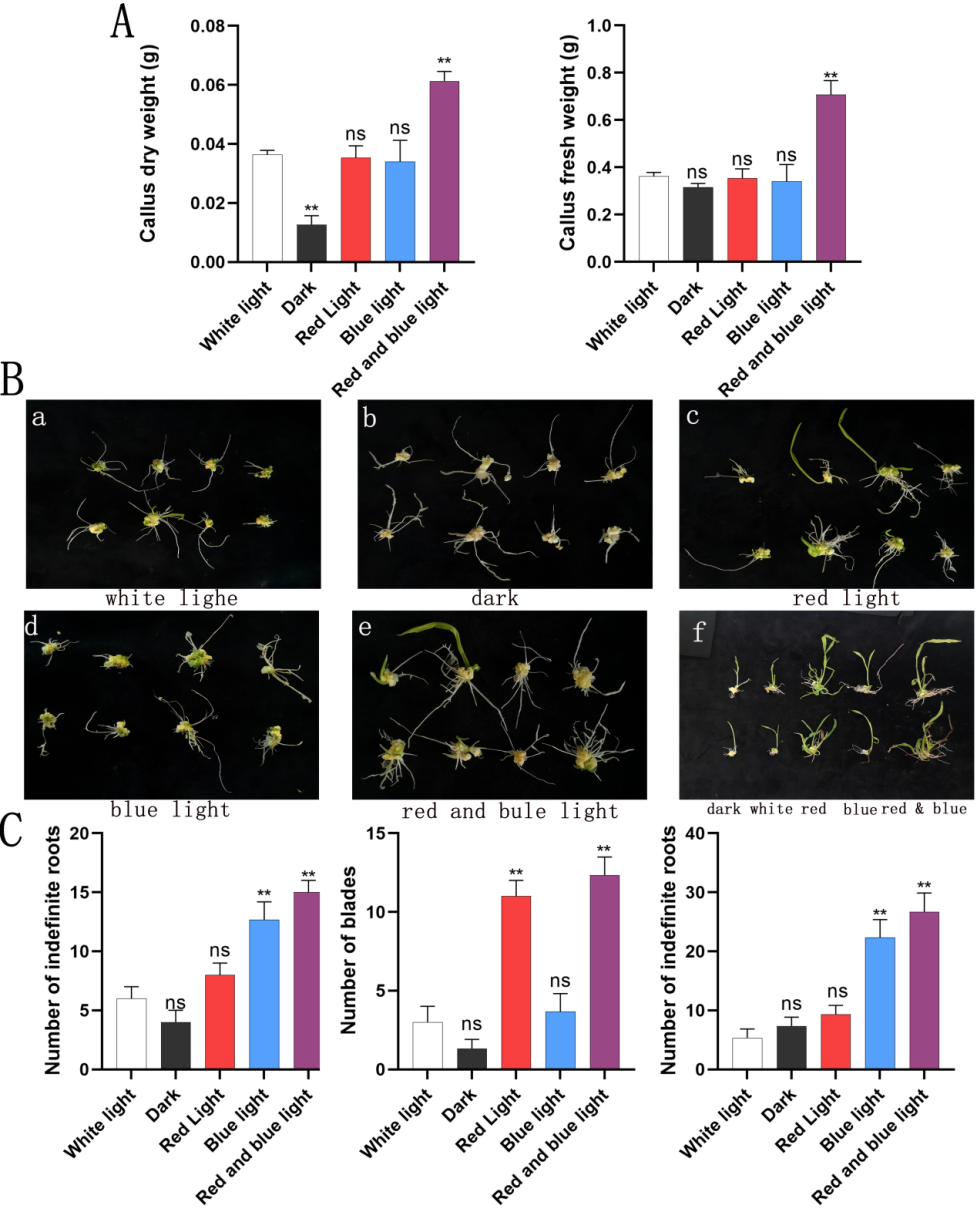
**

**Figure S4.** The growth of callus under different light treatments. (A) Changes of dry weight and fresh weight of callus in seven days.In all experiments, the changes of biomass under white light irradiation were the control group; (B) Callus differentiation under different light treatments, (a)white light→darkt→(c)red light →(d)blue light→(e) red and blue light;(f)Phenotypic map of callus differentiation on the fifteenth day, left→right, dark→white→red→blue→red and blue. (C) Statistics of leaf and adventitia growth of callus under different light sources; The growth under white light irradiation was the control group. Using Student’s t-test, asterisks indicate statistically signifcant diferences (* p <0.05; ** p <0.01). Data are shown as mean ± SD from three independent experiments.


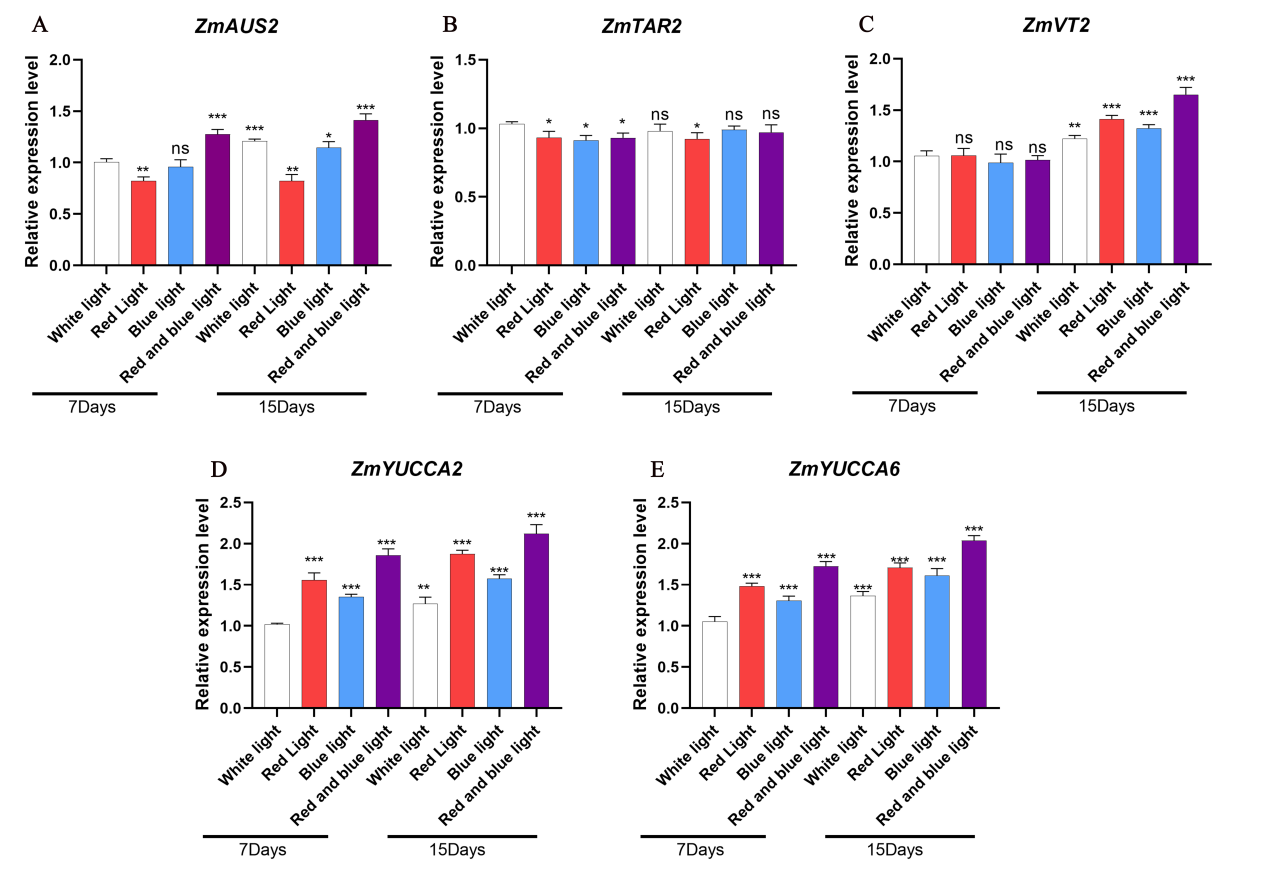


**Figure S5.** Expression of auxin synthesis genes. In all experiments, the expression of genes related to callus cultured under white light for 7 days was the control group.Using Student’s t-test, asterisks indicate statistically signifcant diferences (* p <0.05; ** p <0.01). Data are shown as mean ± SD from three independent experiments


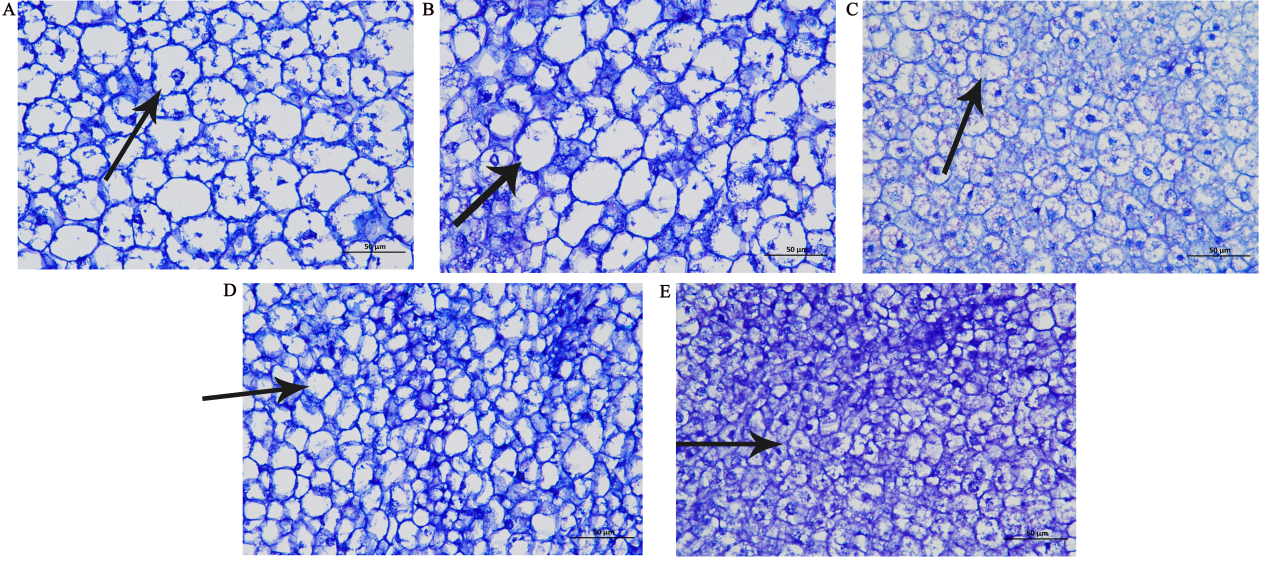


**Fig S6.** Cell sections of callus grown for 15 days under different light sources. (A) Red and blue light; (B) Red light; (C) blue light; (D) white light; (E) dark


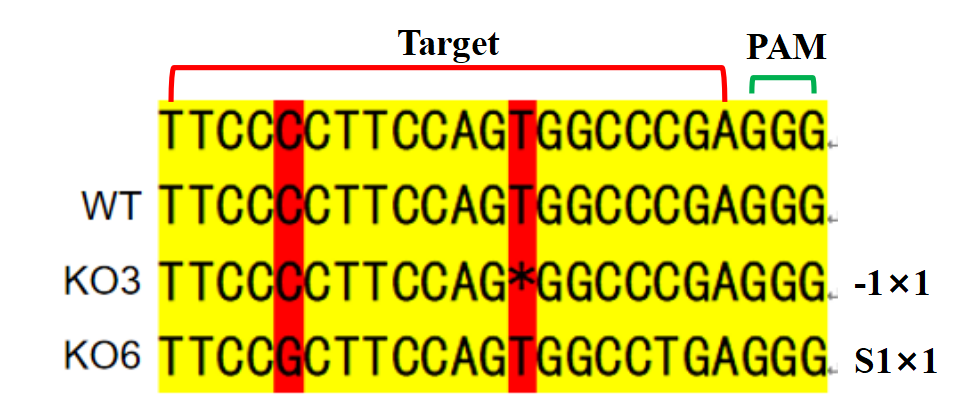


Figure S7. Target mutation information of ZmGRAS46 in maize gene edited plants. Sequences analysis revealed efffcient targeted genome editing using the CRISPR-Cas9 system. The red letters in the sequence indicated mutations, where S indicated replacement, + indicated addition or insertion, and /− indicated deletion. The letter on the right indicated the number of transgenic plants that were mutated in the same type of transgenic line.

**Table S3.** Names and sequences of primers used in the experiments

| Primer name | primer sequence(5’→ 3’) |
| --- | --- |
| ZmGRAS46-F | GGCACCTAGCCGCCCTCTC |
| ZmGRAS46-R | ACCGAAGGAATTTACAGTAAAGTAG |
| ZmGRAS46-3301F | actcttgaccatggtagatctGGCACCTAGCCGCCCTCTC |
| ZmGRAS46-3301R | ggggaaattcgagctggtcaccACCGAAGGAATTTACAGTAAAGTAG |
| q-ZmGRAS46-F | CACTCTGCTGACCGCTTGAC |
| q-ZmGRAS46-R | TCACGTCGTCGAGAACAGGAT |
| q-ZmActin1-F | ATGTTTCCTCCCATTGCCGAT |
| q-ZmActin1-R | CCAGTTTCGTCATACTCTCCCTTG |
| q-ZmFT-F | CTTGCACTGGATGGTGACAGATA |
| q-ZmFT-R | TCAACTGCCTTGTCGTGTTTATG |
| q-ZmLFY-F | AGCACCCCTTCGTGGTCACAGAG |
| q-ZmLFY-R | TACAAAGGTGACCAACCAGGTGTTC |
